# *QCMI*: A method for quantifying putative biotic associations of microbes at the community level

**DOI:** 10.1002/imt2.92

**Published:** 2023-02-16

**Authors:** Xu Liu, Yu Shi, Teng Yang, Gui‐Feng Gao, Haiyan Chu

**Affiliations:** ^1^ State Key Laboratory of Soil and Sustainable Agriculture, Institute of Soil Science Chinese Academy of Sciences Nanjing China; ^2^ University of Chinese Academy of Sciences Beijing China; ^3^ State Key Laboratory of Crop Stress Adaptation and Improvement, School of Life Sciences Henan University Kaifeng China

## Abstract

A workflow has been compiled as “qcmi” R package—the *q*uantifying *c*ommunity‐level *m*icrobial *i*nteractions—to identify and quantify the putative biotic associations of microbes at the community level from ecological networks.

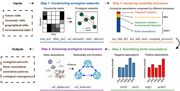

## INTRODUCTION

Microbes are pervasive and play key roles in mediating ecosystem functions [1, 2]. Generally, the wide distributions of microbial organisms are a complementary function of neutral processes, environmental selection, and biotic interactions [3, 4]. Although numerous studies have explored the importance of environmental selection and neutral processes (e.g., dispersal limitation) on microbial communities [5, 6, 7], few have investigated the role of microbial interactions in driving complex microbial community dynamics. Characterizing this interplay is important, as it also contributes to biodiversity and ecosystem functions [8]. Comprehending the various assembly processes, particularly involving unheeded biotic factors, has considerable ecological meanings for real‐world applications, such as fertilizer regulations and biodiversity conservation [9].

Different types of biotic associations between microbes have been identified, with implications for organisms and ecosystems [10, 11, 12]. For example, resident microbial communities have been shown to cooperate to resist invasion by plant‐associated pathogens, suggesting that the strength of facilitative relationships between native microbes can be a good predictor of invasibility and biodiversity for complex systems [13]. On the other hand, antagonistic relationships (e.g., amensalism, exploitation, and competition) among pairs of bacteria were found to prevail in either simple (e.g., guts) or complex (e.g., soils) systems, which may provide new insight for finding antibiotic substitutes, tightly associated with microbial functions [14]. In general, these studies explored microbial interactions by coculturing in the microcosms, using gene copy numbers or microbial biomass to characterize the types of relationships (i.e., positive or negative). However, the studies mentioned above mainly focused on the taxon–taxon relationships, merely involving one or several pairs of microbes (i.e., at the taxon level). There are still a lot of knowledge gaps with respect to the biotic associations of microbes at the community level, especially in the big data era, where large amounts of amplicon‐sequencing‐based community data are being generated with the booms of relevant technology [15, 16]. Thus, proposing a reliable method for quantifying microbial community‐level biotic associations (i.e., implying potential microbial interactions) will aid in understanding the dynamics of microbial communities under contrasting conditions, and ascertain the ecological consequence of biotic interactions on maintaining biodiversity and mediating ecosystem functions.

Network analysis is a promising tool for exploring microbial ecological associations, as it is a reliable mathematical representation in which the nodes and edges represent microbial taxa and associations, respectively [17, 18]. However, it also presents various challenges with respect to quantifying microbe biotic associations at the community level. To begin, popular network methods generally rely on correlations (e.g., *Pearson* and *Spearman*) or covariances (e.g., *SparCC* and *Spiec‐Easi*), and thus, tend to characterize microbial coexistence or co‐occurring patterns (ecological associations rather than biotic associations), which are comprised of multiple assembly processes [19]. This setup makes it difficult to directly represent biotic associations between microbes due to that the patterns may be substantially determined by the failure of certain species to reach an available site (dispersal limitation) and by their shared (or unshared) tolerances to the local set of abiotic conditions (environmental selection). In addition, general network topologies are difficult to characterize complex microbial communities in isolation accurately. Examples include the proportion or frequency of negative links that describe the contribution of antagonistic relationships throughout the whole community and modularity that represents the network architecture between and within different microbial groups [20]. These topologies are typically described at the level of taxa (e.g., degree and betweenness) or network (e.g., modularity and nestedness) and are not specifically designed for microbial data. Using these topologies by themselves to interpret the interacting complexity associated with microbial communities also often poses difficulties. Finally, while several methods can be used to perform detailed sequencing‐based community data analysis—for example, EasyMicroPlot [21], microeco [22], and ggClusterNet [23]—these packages are designed around network inference and layout, and are unable to quantify community‐level microbial associations easily.

**Figure 1:**
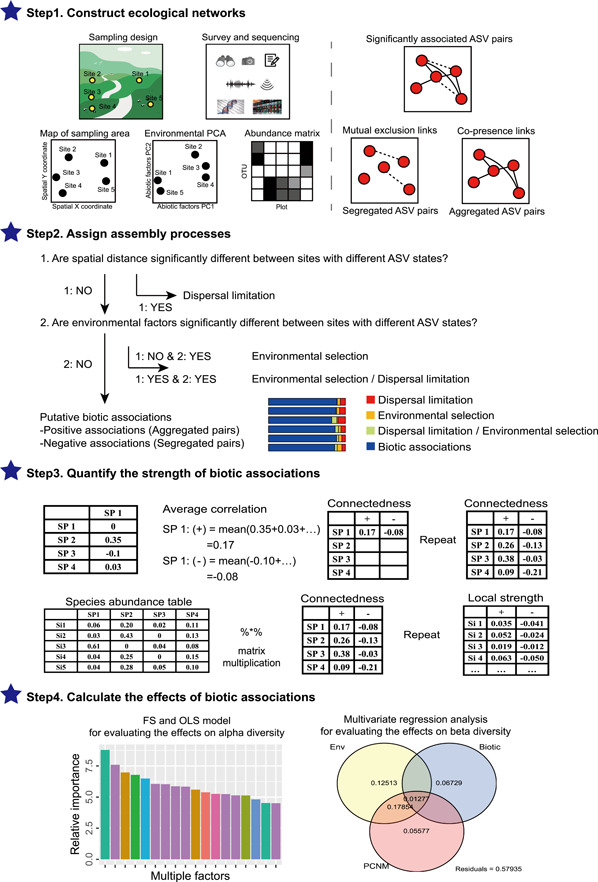
Conceptual framework used to assess putative biotic associations of microbial communities. *Step* 1. Construct ecological networks for sequencing‐based microbial communities. *Step* 2. Assign the assembly processes to each significantly paired ASVs. *Step* 3. Quantify the strength of putative biotic associations at the community level. *Step* 4. Calculate the effects of putative biotic associations of microbes on alpha and beta diversity. ASV, amplicon sequence variant; Env, environment; FS, forward selection; OLS, ordinary least squares; OTU, operational taxonomic unit; PC, personal computer; PCA, principal component analysis; PCNM, principal coordinates of neighbour matrices; SP, species.

Herein, we designed a workflow to quantify putative biotic associations between microbes (i.e., implying potential microbial interactions) at the community level (Figure 1). First, reliable ecological networks were constructed, after which some abiotic‐driven associations, that is, dispersal limitations and environmental selections, were filtered out by link tests. This ensured that the filtered networks represented putative biotic associations as far as possible. Second, the average associating strength was weighted with corresponding microbial abundance, and the resulting metrics for each sample were quantified to the community level to describe the complexity of microbial associations. After verifying that the metrics improved the application methodology for interpreting and predicting microbial communities, and confirming that this method offers a simple way for microbial ecologists to explore the importance of community‐level biotic associations in their study systems, the workflow was developed as a flexible R package (https://github.com/joshualiuxu/qcmi). Finally, this workflow was applied to an empirical data set and used to explore patterns of positive and negative associations across different habitats and their relationships with microbial biodiversity. The *q*uantifying *c*ommunity‐level *m*icrobial *i*nteractions (*qcmi*) package circumvents challenges related to quantifying putative biotic associations at the community level, as well as known flaws associated with microbial community analysis. This is a novel method as it does not rely on the topology description for ecological network structure but instead focuses on the community complexity of microbial associations, thereby exploring the linkages of potential microbial interactions with biodiversity and ecosystem functions.

## METHODS

The package's core processing workflow is presented in Figure 1. The four steps below highlight the package's main functions and characteristics, which were specifically designed to address the aforementioned challenges and flaws.

### Step 1. Constructing ecological networks

First, the input files involved in the workflow include a feature table (i.e., operational taxonomic unit [OTU] table), taxonomic table, geographical table, and environmental table. Ecological networks were inferred using amplicon‐based community data (OTU table), which consists of three functions: *trans_ps()*, *filter_ps()*, and *cal_network()*. The *trans_ps()* function converts the OTU table (relative abundance) and the corresponding taxonomic information into the *phyloseq* format, which enables the user to customize the types of data included in the analysis. This conversion is necessary to resolve challenges associated with inconsistent data requirements for different types of microbial data analysis. Consequently, in addition to community and taxonomy data—metadata, phylogeny, and environmental variables can also be integrated into *phyloseq* format for subsequent analysis.

An important prestep in network analysis is filtering the community data. Although rare species play a pivotal role in the overall microbial community, correlations between low‐abundance taxa (i.e., phylotypes or ASVs) may be biased; thus, we discard low‐abundance taxa to increase the ecological network reliability. The *filter_ps()* function utilizes *phyloseq* for filtering data by both ubiquity and abundance. On the basis of experience and published studies, we generally recommend retaining taxa with >25% occurrence throughout the whole community and a relative abundance of ≥0.001% [1, 19]. Because the output file is still in *phyloseq* format, it can be easily imported for the next step of network inference. Note: If the community data was manually filtered, this step should be ignored.

The *cal_network()* function was applied to construct ecological networks. Two types of network methods are available to perform this task, including correlation and covariance. The correlation method relies on the *corAndPvalue* function in the WGCNA package. The optimal correlation coefficient normally uses a higher filtering criterion (e.g., >0.6). It is worth noting that the correlation‐based approach has its own drawbacks, including the above‐mentioned difficulty in dealing with zero‐inflated models (ignoring rare taxa in the community) and filtering for weakly correlated relationships (e.g., commensalism and amensalism). The other approach is SParse InversE Covariance Estimation for Ecological Association Inference (Spiec‐Easi), which assumes conditional independence, and uses inverse covariance to infer the associations [24]. Spiec‐Easi has gained much recognition for dealing with reliable network associations and is more suitable to deal with the zero‐inflated model due to the global consideration of the associations between different taxa. Although the correlation‐based approach is a useful tool for measuring ecological networks in many contexts, it is a pairwise metric and is limited in a multivariate setting. In contrast, Spiec‐Easi's estimate of entries in the inverse covariance matrix depends on the conditional states of all available nodes, which helps avoid the detection of indirect network interactions. Users need to evaluate the choice of the method based on computational requirements and data volume.

Besides, the methods of random matrix theory (RMT) and iDIRECT were also provided to filter ecological associations, which was described in the GitHub tutorial [17]. The method of RMT enables the association strength to comply with the Poisson distribution in accordance with the laws of nature, while iDIRECT addresses the three main problems, which may exist in original ecological networks, of ill‐conditioning, self‐loop, and interaction strength overflow. We recommend the correlation‐based approach in combination with the filtering network approach of RMT or iDIRECT, rather than adopting the network analysis of Spiec‐Easi.

### Step 2. Assigning assembly processes

The second challenge was determining how to derive biotic associations from complex ecological networks. Positive and negative associations between microbes are a key outcome of community assembly from regional species pools in the ecological networks. Besides, the ecological networks are also determined by the failure of certain species to reach an available site (dispersal limitation) and by their shared (or unshared) tolerances to the local set of abiotic conditions (environmental selection). This required assigning the inferred associations into different types of assembly processes—including dispersal limitation, environmental selection, the overlap of dispersal limitation and environmental selections, and biotic interactions to infer nonrandom associations from ecological networks. The *assigned_process()* function, which incorporates the *test_link_env()* and *test_link_dl()* embedded functions, is a tool for calculating the relative contribution of assembly processes to microbial ecological associations.

Specifically, sequential multivariate analyses (SMAs, also called link tests) were performed by adding spatial distance and environmental dissimilarity and used to assign the processes to pairs of significantly associated taxa. The first step was establishing whether the abundance of significantly associated paired taxa correlated with geographic distance. If yes, then the association was classified as a dispersal limitation. If no, testing continued to assess whether the abundance of pairs correlated with an environmental dissimilarity. If so, the association was classified accordingly. Finally, the resident associations that were not correlated with either space or environment were classified as biotic associations. Since the biotic associations are obtained by exclusion (i.e., decision tree), there is no way to guarantee they were not influenced by other factors or ecological drifts; thus, they are referred to as “putative biotic associations.” The SMAs method served as the Mantel test or multiple regression model to evaluate the relationships between the abundance of pairs and spatial or environmental distance. Note that if the integrated environmental matrix is used (i.e., all the variables were applied to form one dissimilarity matrix), strict covariance detection is required (Spearman *ρ* values <0.5 in this study).

### Step 3. Quantifying biotic associations

The third step was ascertaining how to quantify the strength of putative microbial biotic associations at the community level. Understanding the complexity of microbial associations is the theoretical base in this step to identify and compare positive and negative associations (i.e., on behalf of potential facilitation and competition) across contrasting environments. This required several fundamental functions, such as *zero()*, *pos()*, and *neg()*. The number of zeros was calculated using *zero()*, while *pos()* and *neg()* were applied to calculate the average value of all positive and negative connectedness for each taxon, respectively. Furthermore, using a *qcmi()* core function, the species (corresponding to ASVs or phylotypes) associations (connectedness) weighted corresponding abundance was quantified to the community level via matrix multiplication. Specifically, the positive and negative associations among the taxa were separately recorded, then these values were averaged as the positive and negative connectedness. The strength of putative biotic associations at the community level was obtained by multiplying the abundance table by the positive and negative connectedness, respectively. Thus, there was both positive and negative result. In subsequent analyses, positive and negative associations were regarded as biotic factors that represented the strength of putative biotic associations. In this way, a reliable community‐level topology of positive and negative associations was obtained for subsequent analysis, for example, the patterns of biotic associations between different habitats.

### Step 4. Assessing ecological consequence

Finally, a standard method that included the *cal_alphacon()* and *cal_betacon()* functions was compiled to explore the ecological consequence of biotic associations between microbes on biodiversity, as well as functions. There has been extensive prior empirical evidence that biotic interactions are part of community assembly for microbes, determining microbial biodiversity and function [25, 26, 27]. For example, as stress increases, microbes tend to cooperate rather than compete to maintain biodiversity and metabolic functions to resist environmental stresses [28]. Here, ecological consequences are defined as direct or indirect effects of putative biotic associations of microbes on biodiversity and mediated functions, especially when encountering stress or environmental changes. This step was implemented to increase the fluidity of the overall analysis and to facilitate directly establishing the linkages between biotic factors and biodiversity. The *cal_alphacon()* function contains forward selection and ordinary least squares models for all variables containing abiotic and biotic variables, while the *cal_betacon()* function determines the effect of these factors on community turnover using the Mantel test. Due to the method's feasibility and flexibility, user‐specific data can be imported in each step. Thus, using quantitative metrics results, the user can directly perform an independent analysis that is not limited to these two functions.

## RESULTS AND DISCUSSION

This workflow was applied to empirical data obtained from bacterial communities flourishing in a set of alpine wetlands. The sampling fields displayed a wide range of salinity that was categorized into three types of habitats (i.e., freshwater, brackish, and saline wetlands), representing low, medium, and high salinity stress according to the stress criterion [29]. The goal of this example was to: (i) explore the changes of biotic associations within the bacterial communities across different stress conditions, (ii) correlate microbial biotic associations with biodiversity, and (iii) compare the explanatory rate of biodiversity (alpha and beta) variation with the addition of such biotic factors. In addition, we also explained the results obtained from the qcmi method involved in each step.

**Figure 2:**
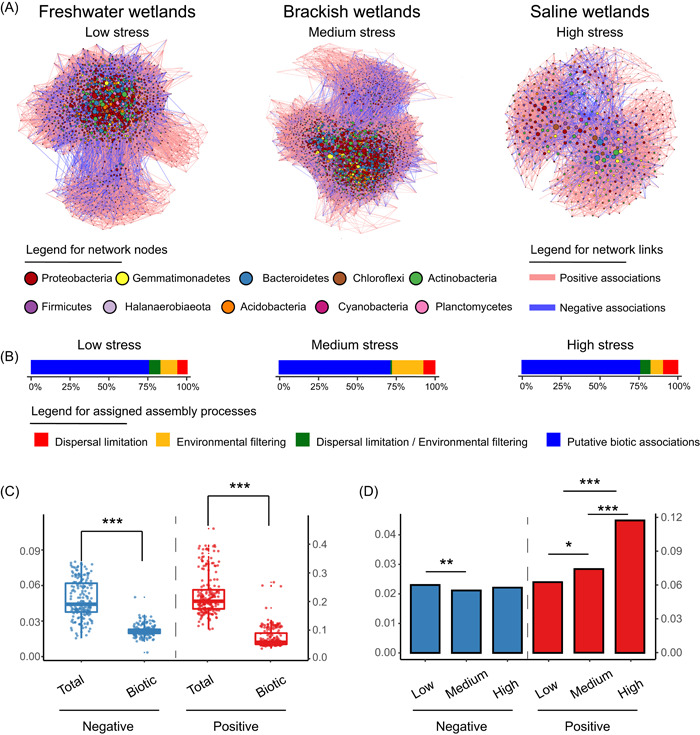
Inference of putative biotic associations for the microbial communities under different habitats of freshwater, brackish, and saline wetlands. (A) Ecological networks were displayed in the Fruchterman–Reingold layout. Nodes indicated major taxa and links indicated ecological associations between nodes. (B) Assigned assembly processes to each significantly paired ASVs were shown via bar plots, including dispersal limitation, environmental filtering, the overlap of dispersal limitation and environmental filtering, and putative biotic associations. Colors represented different processes. (C) Comparisons in negative and positive associations of bacterial communities between original ecological networks (total) and filtered biotic networks (biotic). Student's *t* test was used to detect the differences. (D) The patterns of putative biotic associations within the microbial communities across low, medium, and high stress. ***Represented *p* < 0.001 and **represented *p* < 0.01. ASV, amplicon sequence variant.

As dictated by the above method, the bacterial communities' ecological network was inferred using the Spiec‐Easi method and was comprised of 3195 nodes. The subnetworks were extracted based on the microbial composition within different habitats. The extracting networks were classified into freshwater habitat (nodes = 1244, links = 31,533), brackish habitat (nodes = 2030, links = 67,184), and saline habitat (nodes = 535, links = 5285) (Figure 2A). Before beginning the pipeline, an attempt was made to establish the relationships between microbial diversity (richness) and network topology. Such topology included the number of negative links, modularity, and nestedness, which are reported to be important for ecological communities and functions in previous studies. Results showed that richness was not explained by either the negative links' frequency (*r* = 0.05, *p* = 0.83) or modularity (*r* = –0.02, *p* = 0.94), which is consistent with previous findings from studies encompassing plants to microbes [30, 31]. Although biodiversity depicted a negative correlation with nestedness (*r* = –0.59, *p* < 0.001), these results demonstrate that certain macroscopic network properties (especially at the network level) in isolation have difficulty interpreting and modeling the complex microbial community dynamics.

Next, the contributions of assembly processes to the ecological networks were calculated to obtain putative biotic associations between microbes from total ecological associations (Figure 2B). Here, we selected salinity, mean annual precipitation, soil carbon, soil nitrogen, and soil pH as environmental variables based on the prefitted model, and geographical distance and elevation as dispersal limitation variables for respective link tests (environmental selection and dispersal limitation). The results showed that the percentage of links belonging to putative biotic associations within ecological networks were 76.0%, 70.1%, and 75.5% across freshwater, brackish, and saline wetlands, respectively, after the exclusion of the role of environmental selection and dispersal limitation.

Subsequently, the significant positive and negative associations in the putative biotic networks were collated, and the resulting metrics were obtained by performing matrix multiplication between the abundance and associations of the quantitative results at the community level. A size comparison between the total ecological networks and the biotic networks for the quantified results is shown in Figure 2C. Notably, filtered biotic networks were significantly different from the total ecological networks. These results suggest that previous misuse of co‐occurrence relationships as a direct representation of biotic associations might result in different and even contrasting data. The biotic associations, including those that were negative (blue) and positive (red), also showed different patterns across all habitats on the three levels of stress (Figure 2D). Negative associations differed significantly only between low and medium stress, while positive associations increased and differed significantly with increasing stress, implying that bacteria showed associational resistance by increasing facilitation and cooperation in the face of salinity stress [32]. The inconsistent patterns varying across different stress conditions also indicate the need for screening ecological networks and a simple way to quantify the biotic associations between microbes.

**Figure 3:**
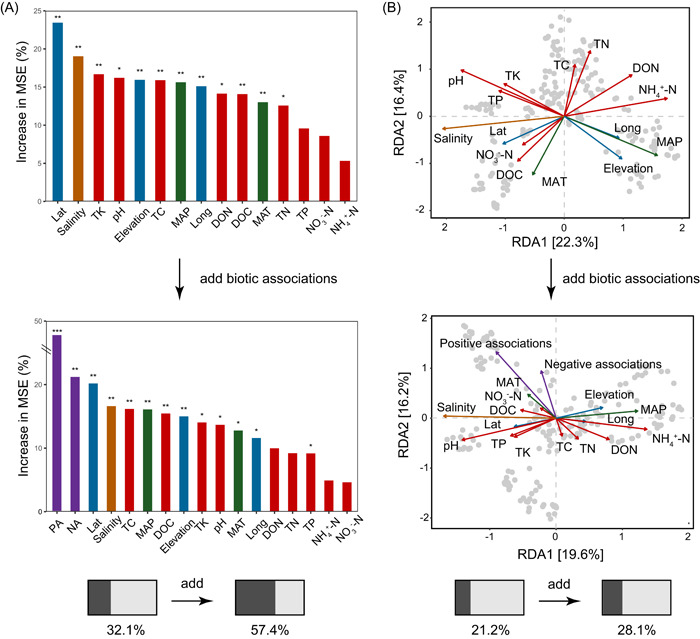
Effects of putative biotic associations on microbial alpha‐ and beta‐diversity. (A) Random forest (RF) predicted the importance (percentage of increase of mean square error [MSE]) of drivers without and with biotic factors for species richness of bacterial communities. (B) Distance‐based redundancy analysis (dbRDA) ordination plotted the relationships between drivers without and with biotic factors and community dissimilarity of bacterial communities. The bottom bar showed the change in the change of explanation. Significance was expressed as ***p < 0.001; **p < 0.01; *p < 0.05. Lat, latitude; Long, longitude; MAP, mean annual precipitation; MAT, mean annual temperature; NA, negative associations; PA, positive associations; TC, total C; TN, total N; TK, total K; TP, total P.

Then, we further explored the relationships between biotic associations and biodiversity (Figure 3). The significance was detected. The results showed that for both alpha and beta diversity, positive associations provided more explanatory variation than negative associations, corresponding to our expectation (i.e., associational resistance). Bacteria may enhance positive relationships between taxa in the face of stressful environments to maintain biodiversity and ensure basal energy metabolism levels [32]. Since the focus of this exercise was to apply the quantitative process, no further investigation into this issue was conducted. Furthermore, explanations describing the difference between predictors with and without biotic associations were compared. Abiotic factors, that is, environmental and space variables, were integrated with the quantified biotic factors to concurrently evaluate their contributions to the diversity patterns. Most variations in bacterial richness were significantly explained by the spatial factor (latitude) to positive associations (Figure 3A). The alpha diversity explanation rate also considerably increased from 32.1% to 57.4%. In addition, the distance‐based redundancy analysis (dbRDA) was applied to study the effects of beta diversity on bacterial communities (Figure 3B). The relative importance of the variables is indicated by the arrow length, which suggests that the most important factor shifted from salinity to positive associations. Overall, the explanation rates by different variables improved from 21.2% to 28.1%.

In previously published studies, microbial ecological networks were widely used to infer ecological associations between pairs of co‐occurring taxa, but often drastically misused as microbial interactions [16, 19]. This study built a general workflow for quantifying biotic associations that avoids the exaggerated description of ecologically meaningless associations, and thus, disentangles assembly processes in significant ecological associations. Every step in the workflow is well documented in previous studies (#1, Spiec‐Easi [24]; #2, Goberna's method [33]; #3, Cohesion [34]; #4, microeco package [22]), making this effort a process‐based as opposed to technology‐based innovation. Recently, Xiao et al. [35] employed Copula‐based addition to improve the overflow problem of network interaction strength, introduced the matrix to eliminate the self‐looping paths that result from indirect paths and obtained a direct interaction network for complex systems through a nonlinear solver and iterative calculations. While they explored the “true interactions” in microbial correlation networks to dismantle direct and indirect relationships, the workflow discussed herein focuses on the “real interactions” among microbes from an ecological perspective, based on community assembly theory, and excludes the co‐varied effects of environmental variables and geographic distances (i.e., abiotic‐driven).

## CONCLUSION

Overall, *qcmi* is a user‐friendly and reliable workflow for quantifying putative biotic associations of microbes at the community level. The package features: (i) the ability to insert selected data and/or variables at each step, thus enabling a customized approach for specific, individualized use; (ii) assembly processes assignment to each significant association, which helps depict true biotic interactions; and (iii) use of matrix multiplication for quantifying the nature of biotic associations from the taxon to community level. Furthermore, testing with empirical community data demonstrated that *qcmi* effectively removes abiotic‐driven associations and significantly improves diversity variation explanations. This methodology circumvents and resolves known challenges and flaws in microbial analysis related to quantifying microbial biotic associations. Thus, this workflow is expected to have broad applications in many fields across contrasting biomes and environments.

## AUTHOR CONTRIBUTIONS

Haiyan Chu and Xu Liu conceived the ideas and designed the methodology. Xu Liu created the supportive scripts and compiled the package. Xu Liu, Yu Shi, Teng Yang, Gui‐Feng Gao, and Haiyan Chu wrote the manuscript.

## CONFLICT OF INTEREST STATEMENT

The authors declare no conflict of interest.

## Supporting information

Supporting information.

## Data Availability

Supporting Information data are available at the manuscript online. The codes have been uploaded and updated in the GitHub website (https://github.com/joshualiuxu/qcmi), as well as in the Gitee website (https://gitee.com/joshllxxxx/qcmi).
